# Malaria in pregnancy complications in Southern Venezuela

**DOI:** 10.1186/s12936-021-03728-9

**Published:** 2021-04-15

**Authors:** Mariestéfany Romero, Elízabeth Leiba, Fhabián S. Carrión-Nessi, Diana C. Freitas-De Nobrega, Serris Kaid-Bay, Ángel F. Gamardo, Melynar Chavero, Luisamy Figuera, Natasha A. Camejo-Ávila, María V. Marcano, Mary Lopez-Perez, David A. Forero-Peña

**Affiliations:** 1 “Dr. Francisco Battistini Casalta” Health Sciences School, University of Oriente – Bolivar Nucleus, Ciudad Bolivar, Venezuela; 2 Biomedical Research and Therapeutic Vaccines Institute, Ciudad Bolivar, Venezuela; 3 Gynaecology and Obstetrics Department, “Ruíz y Páez” University Hospital Complex, Ciudad Bolivar, Venezuela; 4 Centre for Medical Parasitology, Department of Immunology and Microbiology, Faculty of Health and Medical Sciences, University of Copenhagen, Copenhagen, Denmark

**Keywords:** Malaria, Pregnancy, *Plasmodium vivax*, *Plasmodium falciparum*, Venezuela

## Abstract

**Background:**

Pregnant women are particularly vulnerable to malaria infections, increasing the risk of maternal–fetal complications, mainly in high-endemicity areas. However, few studies of malaria in pregnancy (MiP) have been carried out in Latin America, a region with low endemicity and transmission of both, *Plasmodium falciparum* and *Plasmodium vivax*. Despite the high malaria burden in Venezuela in the last years, no recent studies of MiP have been conducted. Hence, epidemiological and clinical characteristics of pregnant women with malaria in southern Venezuela are described herein.

**Methods:**

A retrospective study in pregnant women attending at the “Ruíz y Páez” University Hospital Complex, Bolivar state, Venezuela, was carried out between February and October, 2019. Epidemiological, clinical, and laboratory information was analysed.

**Results:**

Thirty-seven out of 52 pregnant women analysed were infected with *P. vivax*. Age ranged between 15 and 39 years, and adolescent pregnancies were common. Malaria infection was diagnosed mainly during the third trimester of pregnancy (63.4%). The distribution of symptoms and signs as well as clinical laboratory values was similar among *Plasmodium* spp. Although uncomplicated malaria was most frequent, 30% (13/52) had severe anaemia. A high proportion of studied women (44%) presented at least one complication during the pregnancy or delivery. Spontaneous abortion was recorded in four women, and three fetal deaths were observed. Six women had preterm delivery without any further complication.

**Conclusions:**

A high prevalence of maternal–fetal complications was found in the studied population, highlighting the requirement for a careful medical follow up during the prenatal check-ups, which should include routinary malaria tests. Preventive measures as distribution of insecticide-treated mosquito net for pregnant women at risk should also be implemented. Those measures can help to reduce the negative impact of malaria on the newborn and mother.

## Background

Malaria continues to be the leading cause of morbidity and mortality in many developing countries. It is estimated that 229 million cases and 409,000 deaths occurred worldwide due to malaria in 2019 [1]. Pregnant women are particularly vulnerable to malaria infections, increasing the risk of maternal–fetal complications, mainly in high-endemicity areas [2]. The burden of malaria in pregnancy (MiP) in the Americas is uncertain, but in 2007 was estimated at least 3 million pregnant women were at risk of malaria infection [3]. Studies carried out in Honduras [4], Brazil [5], Bolivia [6], and Colombia [7–9], suggest a low frequency of MiP cases (~ 10%) contrasting with 27% in Venezuela [10]. A high proportion of severe cases (4–14%) but low mortality (0–0.2%) has also been reported in Brazil, Bolivia, and Colombia [5–7, 9].

The susceptibility to MiP has long been recognized, with endemicity in the region and gravidity as determining factors. In high-transmission areas, primigravidae are at greater risk of infection, whereas the gravidity effect is less marked in low-transmission areas [11] and absent in areas with epidemic malaria [12]. Maternal age is also an independent risk factor for MiP, with higher risk at younger ages [13, 14]. Due to the acquisition of immunity in the early stages of life in hyperendemic and stable transmission areas, many of the infections during pregnancy are asymptomatic [11]. In contrast, in low endemicity and unstable transmission areas, clinical manifestations in pregnancy are frequent, with a high probability of malaria complications [15].

In malaria-endemic areas of Latin America, a high prevalence of maternal–fetal complications has been reported in women infected with malaria, including severe maternal anaemia and hepatic dysfunction [7–10, 16], prematurity, low birth weight, and congenital malaria [5, 17–20]. In the last decade, Venezuela has experienced a political, social, and economic crisis that has impacted the epidemiology of infectious diseases [21] and the Venezuelan government has issued no official data on pregnancies since 2016. The crisis has led to a drastic increase in the number of malaria cases in the country, which had about 57,926 in 2010, rising to over 467,000 in 2019. Deaths also increased considerably during the same period, rising from 53 to 403 [1], accounting for 55% of the reported cases and 70% of malaria deaths in the World Health Organization (WHO) Region of the Americas in 2019 [1]. Three states, Bolivar, Amazonas, and Sucre, reported 90% of malaria cases in the country, with an increase of 55% in MiP cases compared to 2018 [22]. Nevertheless, there is limited clinical and epidemiological information as well as the impact of MiP in the country. A retrospective study was conducted to describe the clinical and epidemiological characteristics of pregnant women with malaria attending at the “Ruíz y Páez” University Hospital Complex, in Ciudad Bolivar, Bolivar state.

## Methods

### Study area

The study was carried out in Ciudad Bolivar, located in the Bolivar state, southern Venezuela, at 54 m above sea level, covering an area of 209.5 km^2^ and an average temperature of 27.7 °C. Ciudad Bolivar has a population of approximately 567,000 inhabitants. In Bolivar state, 70–80% of malaria cases are caused by *Plasmodium vivax*, and 20–30% are due to *Plasmodium falciparum* [21]. Recently, it has been reported that municipalities in Bolivar state have a heterogeneous annual parasitic incidence (API), with some hotspots in the southeast part [23]. For epidemiological week N° 52 of 2016, the API was 101.7 per 1000 inhabitants in this state [24]. The main hospital in the region is the “Ruíz y Páez” University Hospital Complex, an academic hospital attending patients referred from other hospitals.

### Study design and participants

A retrospective study was conducted in all pregnant women with malaria who consulted at the “Ruíz y Páez” University Hospital Complex between February and October, 2019. Malaria diagnosis was performed by microscopy using thick and thin blood smears, but data on parasite density were not available. A clinician resident from the Gynaecology and Obstetrics Department performed the standard clinical evaluation and a detailed physical examination on all women included in the study. A peripheral blood sample was taken for clinical laboratory analysis according to hospital availability. Women were classified as uncomplicated or severe malaria cases according to the WHO [25] and “Ministerio del Poder Popular para la Salud” (MPPS) of Venezuela [26] criteria, regardless of the malaria parasite species. Pregnant women with uncomplicated and complicated malaria were treated before hospital discharge, according to the recommendation of the health authorities of the Bolivarian Republic of Venezuela [26] and antimalarial drug availability. Briefly, four women infected with *P. falciparum* received quinine (orally, 10 mg/kg thrice a day over 7 days) and clindamycin (orally, 10 mg/kg twice a day over 7 days) and two, artemether plus lumefantrine (orally, twice a day over 3 days). Women infected with *P. vivax* were treated only with chloroquine (orally, 25 mg/kg provided in 3 days), whereas women with mixed malaria were treated with quinine and clindamycin (first trimester) or artemether plus lumefantrine (second and third trimester), as described above. Women with *P. vivax* and mixed malaria were asked to take primaquine after 6 months of breastfeeding. Severe anaemia cases were treated at the hospital with blood transfusion. Intermittent preventive treatment (IPTp) was not provided because it is not included in the Venezuelan national policy. Adolescent pregnancy was defined as a pregnancy in a woman aged 10–19 years [27]. Gestational age was measured from the first day of the last menstrual period. Pregnant women were followed until delivery, and postpartum charts were reviewed to assess the maternal–fetal outcome. After hospital discharge, the women were contacted by phone to know the pregnancy outcome.

### Statistical analysis

Data were analysed using International Business Machines (IBM)® Statistical Package for the Social Sciences® Statistics version 25 (IBM Corp., Armonk, New York, United States) and plotted with GraphPad Prism version 9.0 (GraphPad Software, San Diego, California, United States). Statistical distribution of the data was analysed using Kolmogorov–Smirnov test. Nominal variables were expressed using absolute and relative frequencies, whereas for quantitative variables, measures of central tendency and dispersion were used. Fisher’s exact test was used to compare proportions. Mann–Whitney U test was used to compare two groups. Kruskal–Wallis test was used to compare more-than-two groups followed by pairwise comparison. Median test was used for parameters with extreme outliers. A *p*-value < 0.05 was considered statistically significant.

## Results

### Demographic and epidemiological characteristics

Data from 52 pregnant women with infection by *Plasmodium* spp. were analysed. Most of the women were infected with *P. vivax* (37; 71.2%) and only six (11.5%) with *P. falciparum*. Mixed infection, *P. vivax* and *P. falciparum*, was found in nine (17.3%) women (Table 1).

**Table 1:** Socio-demographic characteristics according to *Plasmodium* spp.

Characteristics		Total, *n* = 52	*P. vivax*, *n* = 37	*P. falciparum*, *n* = 6	Mixed infection, *n* = 9	*p*-value^a^
		Median (min–max)				
Age (years)		22 (15–39)	20 (15–39)	29 (20–31)	25 (17–39)	0.07
Number of pregnancies		3 (1–12)	2 (1–8)	4 (1–5)	3 (1–12)	0.006^b^
Gestational age (weeks)		31 (8–40)	31 (8–40)	32 (16–40)	27 (8–40)	0.85
Number of controls		4 (0–9)	4 (0–9)	4 (0–6)	3 (0–8)	0.87
Previous malaria (number of episodes)^c^		2 (1–33)	2 (1–30)	3 (1–33)	2 (1–30)	0.96^c^

		n (%)	n (%)	n (%)	n (%)	*p*-value^d^
Occupation						
Housewife		35 (67.3)	27 (73)	3 (50)	5 (55.6)	0.4
Merchant		6 (11.5)	3 (8.1)	1 (16.7)	2 (22.2)	0.27
Mineworker		6 (11.5)	4 (10.8)	2 (33.3)	–	0.14

^a^*p*-value using Kruskal–Wallis test

^b^*p-*value using median test, pairwise comparisons showed significant differences between *P. vivax* and *P. falciparum* (*p* = 0.018)

^c^Number of previous episodes was available only for 27 women, *p-*value using median test

^d^*p*-value using Fisher’s exact test

**Fig. 1:**
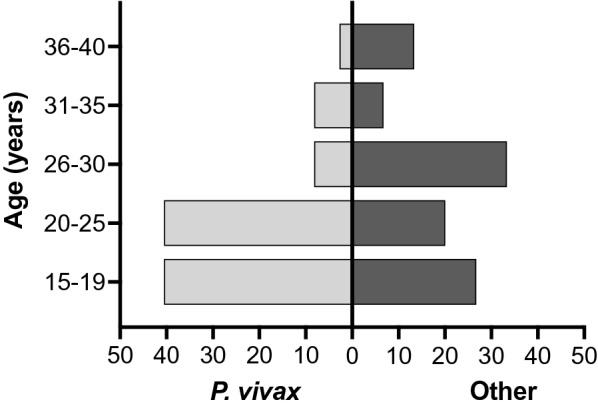
Prevalence and parasite species distribution according to age. Percentage of women infected with *P. vivax* (*n* = 37) or other (*P. falciparum* and mixed infections combined, *n* = 15) at each stratified age group are shown. Adolescent pregnancy: 10–19 years old [27]

Women’s age ranged from 15 to 39 years (Fig. 1); however, 71% of women were ≤ 25 years of age, and adolescent pregnancies were common (17/52). Overall, infections were detected mainly during the third trimester of pregnancy (63.4%). From 27 women self-reporting previous lifetime malaria episodes, 22 were infected by *P. vivax*, and 24 women had the last episode in the previous year. A high proportion of the women were housewives (67.3%) and reached at least primary education, with only two having bachelor degrees (3.8%). Almost half of the women (25/52) were single mother. In 38.5% of patients, the number of prenatal control visits was lower than the recommended by the WHO according to the gestational age [28]. Most of the pregnant women (94.2%) came from the Bolivar state, mainly of Angostura del Orinoco (28.8%), Sifontes (23.1%), Cedeño (11.5%), and El Callao (9.6%) municipality, without significant differences in relation to *Plasmodium* species (*p* = 0.23; Fisher’s exact test).

### Clinical manifestations of MiP

Fever (96.1%), chills (51.9%), asthenia (48%), and headache (32.6%) were the most frequent symptoms. The distribution of symptoms and signs was similar among *Plasmodium* spp., except for headache, which was more frequent in women infected by *P. falciparum* than *P. vivax* or mixed infection (83.3% vs. 27% and 22.2%, respectively; *p* = 0.02; Fisher’s exact test). Diarrhoea, myalgia, and arthralgia were infrequent symptoms, with less than 8% reporting those. The most frequent clinical signs at the physical examination time were fever (86.5%) and pallor (28.8%), with no significant differences between parasite species. Jaundice was found in two women, both infected by *P. vivax*.

### Laboratory findings

Laboratory data at enrolment are presented in Table 2. Data for creatinine and urea evaluation were obtained only from 36 (69.2%) patients, glycaemia in 28 (53.8%), and aspartate aminotransferase (AST) and alanine aminotransferase (ALT) in 13 (25%). Haemoglobin (Hb) levels were analysed in all women (Table 2) and 85% (44/52) of them were diagnosed with anaemia (Hb < 11 g/dL) (Table 3). Hb levels were significantly lower in mixed infection than in *P. vivax* infection (*p* = 0.03; Table 2). The median platelet count was 220,000/µL and only moderate thrombocytopenia (50,000–150,000/µL) was recorded (Table 3). No relevant alterations of liver or kidney function were found. There were no differences in other clinical laboratory levels (*p* > 0.05) according to *Plasmodium* spp. (Table 2).

**Table 2:** Paraclinical findings in pregnant women with malaria

Laboratory parameters	*Plasmodium* spp.				*p*-value^a^
	Total, *n* = 52	*P. vivax*,* n* = 37	*P. falciparum*,* n* = 6	Mixed infection, *n* = 9	
	Median (min–max)				
Haemoglobin (g/dL) (*n* = 52)	9.0 (4–13)	10 (5–13)	7 (5–11)	7.0 (4–10)	0.032^b^
Haematocrit (%) (*n* = 52)	28 (12–39)	30 (14–39)	21 (15–34)	26 (12–33)	0.1
Platelets (×10^3^/µL) (*n* = 51)	222 (93–381)	214 (93–305)	246 (144–298)	238 (170–381)	0.24
Leukocytes (×10^3^/µL) (*n* = 21)	9.0 (3.8–24.8)	9.4 (3.8–24.8)	6.6 (5.4–14.3)	9.00 (5–13.4)	0.43
Glycaemia (mg/dL) (*n* = 28)	78 (54–126)	80 (66–126)	84 (68–95)	73 (54–88)	0.63^c^
Urea (mg/dL) (*n* = 36)	18 (9–52)	17 (11–52)	16.8 (9–25)	22 (15–28)	0.5^c^
Creatinine (mg/dL) (*n* = 36)	0.6 (0.3–1.1)	0.6 (0.3–1.1)	0.6 (0.5–0.8)	0.7 (0.5–1)	1^c^
AST (mg/dL) (*n* = 13)	33 (12–79)	32 (12–45)	–	46 (18–79)	0.28^d^
ALT (mg/dL) (*n* = 13)	18 (10–84)	17 (10–28)	–	31 (13–84)	0.1^d^
Total bilirubin (mg/dL) (*n* = 14)	1.1 (0.4–2.5)	1.3 (0.4–2)	–	0.9 (0.4–2.5)	0.98^d^

*ALT* alanine aminotransferase, *AST* aspartate aminotransferase

^a^*p*-value using Kruskal–Wallis test

^b^Pairwise comparisons showed significant difference between *P. vivax* and mixed infection (*p* = 0.03)

^c^*p*-value using median test

^d^*p*-value using Mann-Whitney U test between *P. vivax* and mixed infection

**Table 3:** Paraclinical alterations in pregnant women with malaria

Laboratory parameters	*Plasmodium* spp.				*p*-value^a^
	Total, *n* = 52	*P. vivax*, *n* = 37	*P. falciparum*, *n* = 6	Mixed infection, *n* = 9	
Hb (*n* = 52)					0.044
Normal Hb (≥ 11 g/dL)	8 (15.4)	8 (21.6)	–	–	
Mild anaemia (9.1–10.9 g/dL)	17 (32.7)	13 (35.2)	2 (33.3)	2 (22.2)	
Moderate anaemia (7–9 g/dL)	14 (26.9)	11 (29.7)	0 (0)	3 (33.3)	
Severe anaemia (< 7 g/dL)	13 (25)	5 (13.5)	4 (66.7)	4 (44.5)	
Haematocrit (*n* = 52)					0.55
Not decreased (≥ 20%)	45 (86.5)	33 (89.2)	5 (83.3)	7 (77.8)	
Decreased (< 20%)	7 (13.5)	4 (10.8)	1 (16.7)	2 (22.2)	
Platelets (*n* = 51)					0.446
Normal (> 150,000/µL)	44 (86.3)	30 (83.3)	5 (83.3)	9 (100)	
Thrombocytopenia (50,000–150,000/µL)	7 (13.7)	6 (16.7)	1 (16.7)	–	
Creatinine (*n* = 36)					0.562
Normal (0.5–1 mg/dL)	34 (94.4)	23 (95.8)	6 (100)	5 (83.3)	
Mild (1.1–1.5 mg/dL)	2 (5.6)	1 (4.2)	–	1 (16.7)	

*ALT* alanine aminotransferase, *AST* aspartate aminotransferase

^a^*p*-value using Fisher’s exact test. Creatinine reference range: 0.4 to 0.8 mg/dL; urea reference range: 5 to 12 mg/dL

### Maternal–fetal complications according to the ***Plasmodium*** spp.

**Fig. 2:**
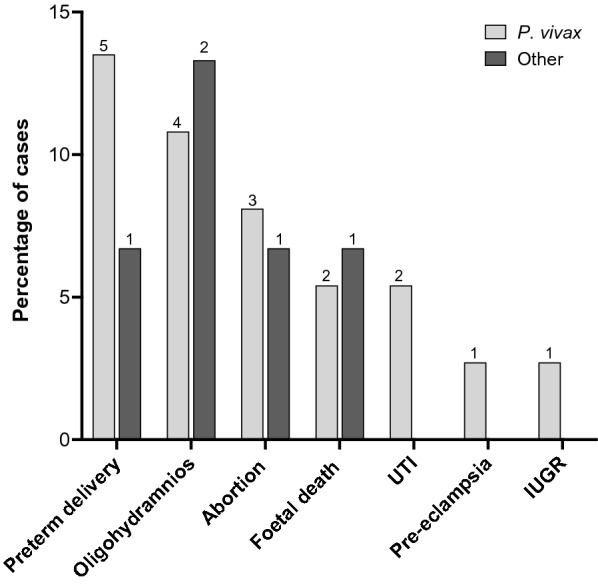
Maternal–fetal complications. Percentage per parasite species and number of cases (above bars) for each complication. For those having more than one complication, only the most important was included. Other, corresponds to cases in women with *P. falciparum* or mixed infection (*n* = 15). *IUGR* intrauterine growth restriction; *UTI* urinary tract infections

Fourteen out of the 52 women (27%) were classified as severe malaria at enrolment, most of them with *P. vivax* infection (11/14; *p* = 0.73; Fisher’s exact test). Twelve women had severe anaemia (Hb < 7 g/dL), one severe anaemia and somnolence, and one more with somnolence as a single criterion. A high proportion (23/52) of studied women presented at least one complication during the pregnancy or delivery (Fig. 2), mainly in those infected by *P. vivax* (18/23; *p* = 0.37). Seven out of those 23 women also had severe MiP. Most of the women with at least one complication were in the third trimester of pregnancy (17/23).

Six women had oligohydramnios; one also presented placental insufficiency and other preterm delivery. Spontaneous abortion was recorded in four women, and three fetal deaths were observed, one also reported uterine rupture and other preterm delivery. A case of pre-eclampsia and another of intrauterine growth restriction were also documented. Two women had urinary tract infections, and six women presented preterm delivery without any further complication. In nine women was not possible to know the pregnancy outcome, including two women with a history of oligohydramnios, one with pre-eclampsia, and another with severe anaemia at the enrolment.

The women with pregnancy or malaria-related complications had a lower number of previous pregnancies (2 vs. 3; *p* = 0.08; Mann–Whitney U test) and a higher number of weeks of gestation (37 vs. 29; *p* = 0.38; Mann–Whitney U test) than those without any complication. Likewise, no significant differences were observed according to age, previous malaria exposure, or time since the last malaria episode. Although 27 women reported previous malaria cases, it is important to notice that only one woman reported malaria in previous pregnancies.

## Discussion

This study describes the clinical and epidemiological characteristics of a cohort of pregnant women with malaria attending an academic hospital in southern Venezuela. Here, the infection by *P. vivax* was the most frequent in agreement with the malaria species distribution in the country [29] as well as with other studies in pregnant women in Venezuela [10, 30, 31], Colombia [19, 32, 33], Brazil [5], and Bolivia [6]. Mixed infections were also frequent, as reported by Morao et al. in Venezuela [34]. In agreement with previous studies in Latin America [9, 32, 34, 35] a high proportion of women were young, with several of them being adolescents, reflecting the fertility rate reported for Venezuela, the highest in Latin America, with 85 births per 1000 adolescents aged between 15 and 19 years old in 2018 [36]. Most of the women were from Angostura del Orinoco and Sifontes municipality as reported previously [10], which perhaps be related to the continuous migration of individuals from the community to gold mining areas, contributing to the malaria transmission [21, 23, 34]. Because of the nature of the study and due to limited access to recent official data on pregnancies, it was not possible to estimate the frequency of MiP. A previous study in the Sifontes municipality reported a malaria incidence of 27% in a cohort of 449 pregnant women [10] and according to WHO/Pan American Health Organization, cases of MiP in the country increased 55% in 2019 [22].

The clinical manifestations were similar to those reported by other authors in Venezuela [10, 30] and Colombia [7, 9, 32]. The high frequency of headache in women with *P. vivax*, together with the high frequency of fever, supports the practice of performing malaria diagnostic tests at prenatal check-ups, favouring timely diagnosis in highly endemic areas as has been suggested before [5, 37]. Indeed, early malaria diagnosis and treatment reduce maternal mortality [38]. Severe anaemia is responsible for around 50% of the MiP complications in endemic areas with intense and stable transmission [34]. In this study, 84.6% of women presented Hb alterations that ranged from mild to severe, with severe anaemia as the most frequent malaria complication among all women (23%), in agreement with studies in Brazil and Venezuela [34, 35], but in contrast with results from Colombia, where mild-to-moderate anaemia and severe anaemia were observed in ~ 66% and ~ 3%, respectively [7, 9].

The most important finding of this study is the high prevalence of maternal–fetal complications (44%), with preterm delivery, oligohydramnios, spontaneous abortion, and fetal death as the most frequent complications. Almost all of them in women with malaria by *P. vivax*, an infection usually considered less severe as compared to *P. falciparum* malaria. This is assumed to be related to the lack of placental sequestration in *P. vivax* infections and the parasite tropism for reticulocytes accounting for a milder form of anaemia [39]. Percentage of spontaneous abortion in *P. vivax* infections was lower than previously reported among hospitalized women (8% vs. 17%) in Venezuela [30] but higher than found in a community study in Bolivar state (3%) [10]. Those results contrast with low percentages of spontaneous abortion reported in Colombia (< 0.5%) [7, 9, 16]. The prevalence of preterm delivery regardless of other complications was higher than reported by other studies (18.6% vs. < 10%) in Venezuela [10, 30], Brazil [40], and Peru [41], but much lower than reported in hospitalized pregnant women from Colombia (71%) [32]. Anaemia has been associated with a higher proportion of preterm delivery, which could explain the high frequency of this complication in the studied population. On the other hand, the percentage of women with oligohydramnios (12%) and intrauterine growth restriction (2%) was lower than documented by studies (40 and 80%, respectively) in Peru [41] and Colombia (12.8%) [20].

Due mainly to logistical and financial constraints, this study had some limitations. First, the clinical and epidemiological characteristics of MiP are described only in a single diagnostic centre. The number of *P. falciparum* and mixed infections is limited, and the comparisons among parasite species should be interpreted with caution. Thus, additional studies are needed to investigate the impact of malaria on maternal–fetal health in different sentinel centres in the country and increasing the sample size. Second, complete paraclinical examinations were carried out only in a subset of the women. Third, the presence of comorbidities and days of illness before the malaria diagnosis is unknown. Finally, the maternal–fetal complications are unknown for some women.

## Conclusions

Malaria by *P. vivax* was the most frequent among pregnant women, in agreement with the distribution of malaria parasite species in Venezuela. A high proportion of women had the last malaria episode in the previous year, and a relapse cannot be ruled out due to the restriction of using primaquine during pregnancy. Studies of relapse in malaria by *P. vivax* in the country should be carried out. The high prevalence of maternal–fetal complications found in the studied population, with a potentially negative effect in the newborn, supports the need for a careful medical follow up during the prenatal check-ups, which should include routinary malaria test for timely malaria diagnosis and antimalarial treatment. Preventive measures as distribution of insecticide-treated mosquito net for pregnant women at risk should also be implemented. Future studies should include complete paraclinical examinations in all women and different health centres in the country.

## Data Availability

All data generated or analysed during this study are included within this article.

## Abbreviations

Hb: Haemoglobin
MiP: Malaria in pregnancy
WHO: World Health Organization
